# 

**Published:** 1781-11

**Authors:** 


### PROMOTIONS.

*Oct.* 26. Mr. John Billam of Trinity college, Cambridge, to be M. B.

*Nov.* 3. Mr. —— Mumforth to be ſurgeon to the 59th regiment of foot, in the room of Mr. John M'Culloch. Mr. —— Girdleſtone to be ſurgeon to the 101ſt regiment of foot, in the room of Mr. —— Allen.—6. Dr. John Daviſon, Dr. Snowden White, and Dr. John Storer, to be phyſicians; Mr. John Hollis, Mr. Thomas Wright, Mr. John Bigſby, and Mr. J. Attenburrow, ſurgeons; and Mr. John Debraw, apothecary to the county infirmary at Nottingham.—8. Dr. Samuel Foart Simmons, to be phyſician to St. Luke's Hoſpital, in the room of the late Dr. Brooke. Dr. Patrick Dugud Leſlie, phyſician at Durham, to be Fellow of the Royal Society.

---

DEATHS.

### DEATHS.

Lately at Rochefter, Thomas Cradock, M. B. aged 84.

——— Naffington, Northamptonfhire, Mr. Forfter, furgeon and apothecary.

——— Hufbands Bofworth, Leicefterfhire, Mr. Robert Heygate, furgeon and apothecary.

*Oct.* 28. At Potton, Bedfordfhire, Mr. Henry Sheffield, furgeon and apothecary.—29. At Canterbury, Mr. Thornton, apothecary.

*Nov.* 7. At Rye in Suffex, Mr. Needler Chamberlaine, furgeon and apothecary.—15. At Deptford, aged 104 years, Mr. John Brickley, formerly an army furgeon.

---

### SECTION IV.

### MONTHLY CATALOGUE.

1. AN Enquiry into the nature, caufes, and method of cure, of Nervous Diforders. In a letter to a friend. By *Alex. Thomfon*, M. D. 8vo. *Murray*, London, 1781. 35 pages. 1s.

This little performance contains feveral judicious remarks on the nature and treatment of nervous diforders. The following extract includes part of the author's obfervations on the means of cure in theſe caſes, and will ſerve as a ſpecimen of the work.

" In proſecuting the radical cure of nervous complaints, the moſt efficacious remedies are ſteel, the Peruvian bark, and the cold-bath. In ſome caſes, the two firſt may with propriety be combined; in others, one of them only can be adminiſtered with advantage. When the pulſe is ſlow, either may be given; if frequent, unleſs this ſymptom be occaſioned by weakneſs, ſteel eſpecially is unadviſeable. It ought, however, to be remarked, that in thoſe diſorders, the pulſe is ſubject to great irregularity; and therefore, that no poſitive indication ſhould be drawn from it, without the concurrence of other circumſtances. Both ſteel and bark are improper while the patient is plethoric, or afflicted with fixt pains in the belly. Chalybeate waters are indicated in the ſame circumſtances with ſteel medicines, and may be found alike beneficial.

By nothing is the body more effectually ſtrengthened than by the cold-bath, which ſhould, therefore, never be neglected, when no particular circumſtance forbids. To bathe three or four times a-week is ſufficient; and at the end of every three months the practice ought to be intermitted for a week or ten days; a caution alſo alſo to be adopted with regard to the ſtrengthening medicines.

"The method of uſing the cold-bath in ſummer and autumn, and the internal corroborants in the winter and ſpring, is liable to exception. For the former, by far the more efficacious, is by this means too long interrupted, and in the ſeaſons when, if the bath be of freſh water, its influence is ſenſibly greater. A more adviſeable method is to uſe the bath, with the bark and chalybeates, in ſhorter alternations; bathing two or three weeks, and then taking the medicines half that time. Nay, there lies no objection againſt the uſe of the Peruvian bark every day the perſon does not bathe; obſerving to intermit the medicine occaſionally for a few days. A light tincture of the bark, joined to bitters, may be uſed in this manner with ſucceſs.

"When acids do not diſagree with the ſtomach, twenty or thirty drops of the elixir of vitriol may be taken once or twice a-day, in a ſmall diſh of camomile or roſemary tea.

"Beſide thoſe remedies, and a ſtrict attention to diet, it is neceſſary that the patient uſe daily exerciſe; than which nothing is more conducive to promote digeſtion, facilitate ſleep, and reſtore to the conſtitution the poſſible degree of ſtrength.

Both

Both the kind and duration of exercife muft be fuited to the ftate of the patient. The moft beneficial is riding on horfeback. Whatever be the exercife, the perfon fhould carefully avoid fatigue, which not only retards the cure, but excites palpitations and fpafms of different parts, with variety of uneafinefs. In thofe circumftances, fetid medicines have peculiar power. Of ufe they may be alfo to allay the craving of the ftomach, to which nervous perfons are frequently fubject. But this fymptom finds, perhaps, no better remedy than a cruft of bread, or any thing folid.

" A dry air, temperately cool rather than warm, is moft favorable to the cure of nervous diforders. If ever the Peruvian bark be preferable to cold-bathing, it is during a moift ftate of the atmofphere. At leaft in fuch feafons it might be alternated with the other. In moift weather, the flefh-brufh is alfo of particular fervice; but it fhould always be adminiftered by an attendant. For a nervous perfon is foon fatigued with the exercife: and as the moft proper time for it is that of going to bed, the confequence of fuch fatigue might be continued watchfulnefs.

" In the treatment of nervous diforders, no care is more important than that of preferving tranquillity.

quillity. Vexation, grief, and despondence are peculiarly injurious; while on the other hand, cheerfulness, and whatever promotes it, as agreeable company and amusement, essentially conduce to the cure; which may be also considerably forwarded by the practice of early rising.

"When the disorder may have resisted all the above remedies, from the use of a milk-diet has relief sometimes been effected. But this final resource must either be continued during life, or quitted with great caution, and by slow degrees."

2. Physiological Disquisitions; or, discourses on the natural philosophy of the elements. 1. On matter; 2. On motion; 3. On the elements; 4. On fire; 5. On air; 6. On sounds and music; 7. On fossil bodies; 8. On physical geography, or the natural history of the earth; 9. On the weather. By *W. Jones*, F. R. S. Rector of Paston in Northamptonshire, and author of an Essay on the first principles of natural philosophy. 4to. *Robinson*, London, 1781, 1l. 1s. in boards.

3. The new British Dispensatory, 12mo. *Newbery*, London, 1781. 3s.

4. Essais historiques, litteraires, et critiques, sur l'art des accouchemens, ou Recherches sur les coutumes, les mœurs, et les usages des anciens et des modernes dans les accouchemens; l'etat des des fages femmes, des accoucheurs, et des nourices chez les uns et les autres: ouvrage dans lequel on a recueilli les faits les plus intereffans et les plus utiles fur cette matiere, avec un grand nombre de notes curieufes, et anecdotes fingulieres. *i. e.* Effays hiftorical, literary, and critical, on the art of midwifery; or, Inquiries concerning the cuftoms, manners, and ufages of the ancients and moderns in midwifery; the ftate of midwives, accoucheurs, and nurfes amongft the one and the other: being a collection of the moft interefting and ufeful facts on this fubject, with a great number of curious notes and fingular anecdotes. By *M. Sue*, jun. late provoft of the college of furgery, and profeffor of anatomy and furgery in the practical fchool, furgeon in ordinary to the Hotel de Ville, and member of the academies of Montpellier, Lyons, Rouen, Dijon, Bordeaux, &c. 8vo. Paris, 1779. 2 vols.

Thefe two volumes, the firft of 600 and the fecond of 731 pages, contain an immenfe quantity of good and bad materials, collected from Aftruc, Haller, Portal, and others. A great part of the work is filled with the author's notes, printed in a fmall type, in which we meet with biographical anecdotes of an infinite number of perfons, persons, both ancient and modern, who do not seem to have the most distant connexion with the subject; such, for instance, are Abraham, Isaac, David, Ezekiel, Origenes, Flavius Josephus, Suetonius, Lycurgus, Drelincourt, Cato, Cicero, Titus Atticus, Horace, Apollonius, Propertius, Servius, Symmachus, Pollux, Paulus Jovius, Tacitus, Nero, Caligula, Homer, Diodorus Siculus, Strabo, Job, Thomas d'Aquinas, Euripides, Demosthenes, Mahomet, Shakespeare, Voltaire, &c. &c. &c.

As a specimen of the state of midwifery in England, he presents his readers with a long extract from Tristram Shandy, and with Dr. Nicholl's petition of the unborn babes.

5. Dissertatio chemica de analysi ferri, quam venia ampliss. facult. philos. preside mag. Torb. Bergman, chemiæ prof. &c. publice ventilandam sistit *Johannes Gadolin*, Aboa-Fenno. 4to. Upsal, 1781. p. 74.

This elaborate and ingenious performance is founded on a great number of experiments, which cannot fail of rendering it extremely interesting to the chemical reader.

6. Histoire des insectes nuisibles à l'homme, aux bestiaux, à l'agriculture, et au jardinage, avec les moyens qu'on peut employer pour les

 detruire, detruire, ou s'en garantir, ou remedier aux maux qu'ils ont pû occaſionner. *i. e.* Hiſtory of inſects hurtful to man, to cattle, to agriculture, or to gardening; with the means that may be employed for deſtroying or guarding againſt them, or for remedying the evils they may have occaſioned. 12mo. Paris, 1781. 339 pages.

This work, which is written by M. Buchoz, ſeems to be a uſeful compilation.

7. Delectus obſervationum practicarum ex diario clinico operâ et ſtudio *Philippi Rodolphi Viat*, M. D. ſoc. reg. ſc. Gottingenſis ſodalis. 8vo. Winterchur, 1780. 318 pages.

8. *J. Frid. Cartheuſeri*, M. D. et profeſſoris, &c. Diſſertationes phyſico-chemico-medicæ de quibuſdam materiæ medicæ ſubjectis exaratæ, nunc iterum recuſæ. 8vo. Francof. ad Viadrum. 1779. p. 168.

Theſe diſſertations are five in number. In the firſt, the author treats of the Mungo root, a ſubſtance that is brought from the Eaſt Indies, and ſold by the Dutch at a high price. To a deſcription of the plant and its virtues, which is taken chiefly from Kempfer, our author has added its chemical analyſis. This root, particularly its cortical part, has a very bitter taſte, not unlike that of the *gentiana lutea.* The author, author, from his experiments, confiders wine as the beft menftruum of this fubftance, and affures us he has frequently feen it efficacious in debility of the ftomach.

In the fecond differtation the author gives a complete hiftory of the balfam of Mecca, in which he points out its chemical analyfis, adulterations, medicinal properties, &c.

In the third differtation Dr. Cartheufer treats of millepedes, of which he gives the chemical analyfis. As thefe infects contain a great quantity of mucilaginous and earthy particles, he fuppofes them to poffefs little or no medicinal virtues, efpecially as he has often given them in cafes in which they are ufually prefcribed, without having experienced the leaft good effect from them.

In the fourth effay we have an account of the chemical analyfis, &c. of Caffia buds; and in the fifth and laft differtation the author treats of the Columbo root. From his experiments with this fubftance, he fuppofes rectified fpirit to be the beft menftruum of it. He has obferved, that vinegar increafes its bitter tafte, and that the *lixivium tartari* has a contrary effect. This leads him to caution us againft combining alkalis with bitters in the cure of difeafes.

9. *J. Frid. Cartheuseri*, M. D. &c. Dissertationes nonnullæ selectiores Physico-chemicæ ac Medicæ, varii argumenti post novam lustrationem ad prælum revocatæ. 8vo. Francofurt. ad Viadrum, 1779. p. 366.

This collection may be considered as a continuation of the preceding article. It contains thirteen dissertations, of which the titles are as follow: 1. *De cinnabaris inertia medica*; 2. *De eximia myrrhæ genuinæ virtute*; 3. *De recta motuum Naturæ existimatione in morbis*; 4. *De oleo kajeput*; 5. *De hydrophthalmia*; 6. *De crocis martialibus*; 7. *De morbis morborum remediis*; 8. *De amylo*; 9. *De suppuratione et tinnitu aurium*; 10. *De incommodis senectutis*; 11. *De noxia retinendorum excretione, et excernendorum retentione voluntaria*; 12. *De respiratione*; 13. *De sale volatili oleoso solido in oleis æthereis nonnunquam reperto*; 14. *De remediis antisepticis*.

Of the concrete substance, which is the subject of his 13th essay, he observes, that it is not, as some have imagined, a species of camphor, but a volatile oily salt. It has been observed in the oils of orange peel, cinnamon, &c. adhering, some months after distillation, in a small quantity, to the sides of the vessel containing them. This substance easily melts upon the tongue, but tastes exactly exactly like the oil it is procured from, and has none of the properties of camphor. He supposes this kind of salt to be similar to that which is obtained from benzoin, styrax, and balsam of Tolu. He adds, that it resembles sugar, and on that account might with propriety be called *saccharum volatile*.

10. Uber die behandlung des venerischen ubels, *i. e.* of the Treatment of the Venereal Disease. By *Charles William Nose*, M. D. 8vo. Augsbourg, 1780. 192 pages.

The author of this work, who is physician to St. Martin's hospital for venereal patients at Augsbourg, confines his inquiries chiefly to the use of sublimate in the cure of the lues venerea. He generally administers this medicine in the form of an electuary by mixing it with rob of elder, or the extract of bark. Being persuaded that the constant effect of mercurials is to weaken the system, he insists on the necessity we are under of combining them with tonics.

11. Dissertatio inauguralis de hydrope. Auctore *J. C. F. Bloedau.* 4to. Wittemberg. 1780. p. 24.

12. Nouvelles observations et recherches analytiques sur la magnesie du sel d'Epsom, suivies de reflexions sur l'union chymique des corps.

*i. e.*

i. e. New obſervations and analytical inquiries relative to the magneſia of Epſom ſalt, to which are added reflexions on the chemical union of bodies. By *Peter Butini*, citizen of Geneva. 8vo. Geneva, 1781.

The principal obſervations in this tract relate to the ſolution of magneſia in water impregnated with fixed air being in greater quantity than in pure water, and more ſo in cold than in hot water. The author has remarked, that a ſolution of magneſia in water impregnated with fixed air is precipitated by heat alone, and that this precipitation forms hexagonal priſms.

13. Ad opuſculum cui titulus eſt, "Queſ-"tionum medicarum ſeries chronologica, &c. "&c." ſupplementa et emendationes ab anno 1508 uſque ad annum 1763. 4to, Paris. p. 9.

The work, to which this is a ſupplement, contains a catalogue of all the theſes maintained in the ſchools of phyſic at Paris, from 1508 to 1763, and a liſt of the members of the Faculty of Phyſic in that city during the ſame period. This ſupplement is given gratis to the purchaſers of the original work.

14. Diſſertatio inauguralis medico-chirurgica de fiſtulam lachrymalem ſanandi methodis. Auctore *Joanne Georgio Schultze*. 8vo. Straſburgh, 1780. p. 80.

15. Differtatio inauguralis medico-chemica de anathymiafi cinnabaris. Auctore *Dionifio Ponyrka*. 4to. Strafburgh, 1780. p. 40.

16. Differtatio fiftens Helminthocorti hiftoriam, naturam atque vires. Auctore *Petro Jofepho Schiwendimann*, 8vo. Strafburgh, 1780. p. 40.

17. Recherches chymiques fur l'etain, faites et publiées par ordre du gouvernement; ou reponfe à cette queftion; Peut-on fans aucun danger employer les vaiffeaux d'etain dans l'ufage economique? *i. e.* Chemical inquiries concerning tin, made and publifhed by order of government: or an anfwer to this queftion; May we without any danger employ tin veffels for domeftic purpofes? By Meffieurs *Boyen*, apothecary major to the army, and *Chalard*, Provoft of the College of Pharmacy. 8vo. Paris, 1781. 285 pages.

The refults of thefe inquiries are, that tin veffels may be employed with fafety for culinary purpofes, and that tin has no deleterious property. The authors obferve, that a great number of plumbers, houfe-painters, and other perfons, who are expofed to the noxious fumes of lead, are every year brought to the Charity Hofpital at Paris with colic, palfy, &c. but on ininquiry they do not find that any workers in tin ever apply there for the cure of any diseafe brought on by that metal.

18. Lettre d'un medecin de la Faculté de Paris à un medecin du College de Londres; ouvrage dans lequel on prouve contre M. Mefmer, que le magnetifme animal n'exifte pas. *i. e.* A Letter from a phyfician of the Faculty of Paris, to a phyfician of the College of London, written to prove, contrary to the affertion of M. Mefmer, that there is no fuch thing as animal magnetifm. 8vo. Hague, 1781. 70 pages.

19. Difquifitio medico-forenfis, quâ cafus, annotationes ad vitam fœtûs neogoni disjudicandam facientes proponuntur. Auctore *Chr. Frid. Joeger*, M. D. and Prof. 4to. Ulm, 1780.

20. Differtatio inauguralis de apoplexia biliofa. Auctore *Joanne Gulielmo Moll*, Colonienfi. 4to. Gottingæ, 1780. p. 40.

21. Differtatio inauguralis de gaftritide. Auctore *Philippo Chriftophoro Bode*, Luneburgenfi. 4to. Gottingæ, 1780. p. 24.

